# Association between Cerebrospinal Fluid Soluble *TREM2*, Alzheimer’s Disease and Other Neurodegenerative Diseases

**DOI:** 10.3390/jcm12103589

**Published:** 2023-05-22

**Authors:** Wenchuan Zhou, Yutong Zhou, Jing Li

**Affiliations:** Department of Ophthalmology, Xinhua Hospital Affiliated to Shanghai Jiao Tong University School of Medicine, Shanghai 200092, China

**Keywords:** soluble *TREM2*, neurodegenerative diseases, Alzheimer’s disease, meta-analysis

## Abstract

Background: Cerebrospinal fluid (CSF) soluble triggering receptor expressed on myeloid cells 2 (s*TREM2*) is a potential biomarker and therapy target for neurodegenerative diseases (NDDs). The purpose of this meta-analysis was to investigate the association between CSF s*TREM2* level and NDDs, and to reveal the dynamic changes in CSF s*TREM2* level in Alzheimer’s disease (AD) continuum. Methods: We systematically searched PubMed, Embase, Web of Science, and Cochrane Library databases for observational studies, which compared the levels of CSF s*TREM2* between NDDs and controls. Sources of heterogeneity were analyzed using sensitivity analysis, subgroup analysis and meta-regression. We assessed pooled data using a random-effects model. Results: Twenty-two observational studies which included 5716 participates were identified. Compared with the controls, the whole AD continuum group showed a significant increase in CSF s*TREM2* level (standardized mean difference [SMD]: 0.41, 95% confidence intervals [CI]: 0.24, 0.58, *p* < 0.001). The mild cognitive impairment (MCI) group displayed the largest effect size (SMD, 0.49 [95% CI: 0.10, 0.88], *p* = 0.014), followed by the AD cohort (SMD, 0.40 [95% CI: 0.18, 0.63], *p* < 0.001). The increase in s*TREM2* in the preclinical stage of AD (pre-AD) group was the lowest (SMD, 0.29 [95% CI: 0.03, 0.55], *p* = 0.031). Other NDDs also showed an increase in the CSF s*TREM2* levels compared with control groups (SMD, 0.77 [95% CI: 0.37, 1.16], *p* < 0.001). Conclusions: The pooled data confirmed that NDDs are associated with increased CSF s*TREM2* level, thereby suggesting the CSF s*TREM2* as a potential dynamic biomarker and therapy target for NDDs.

## 1. Introduction

Triggering receptor expressed on myeloid cells 2 (*TREM2*) is an innate immune receptor. In the central nervous system (CNS), *TREM2* is almost exclusively expressed on the surface of microglia [1]. It is a cell surface single-pass transmembrane glycoprotein with three domains: an extracellular immunoglobulin-like domain, a transmembrane domain and a short cytoplasmic tail [2]. Extracellular ligand binding activates *TREM2* and its adaptor proteins (DNAX activation proteins, DAP10/12), initiates intracellular signaling cascades and affects a series of biological activities, such as cell survival, proliferation, metabolism, cytoskeleton remodeling, and phagocytosis [3,4,5,6,7].

Homozygous loss-of-function mutations in *TREM2* is associated with an autosomal recessive form Nasu–Hakola disease that is characterized by early onset dementia, bone cysts and consequent fractures [8]. Heterozygous rare variants in *TREM2* are associated with increased risk for neurodegenerative diseases (NDDs), including Alzheimer’s disease (AD), Parkinson’s disease (PD), frontotemporal dementia (FTD) and amyotrophic lateral sclerosis (ALS) [9,10,11,12,13]. Studies showed that *TREM2* is needed for stress-induced activation of microglial cells in the brain [14,15]. Such activated microglial cells, also called disease-associated microglia (DAM) are neuroprotective through enhanced phagocytosis in neural degenerative conditions [16]. Due to its important role in microglial cell activation, *TREM2* is extensively studied as a potential therapeutic target for NDDs.

**Figure 1:**
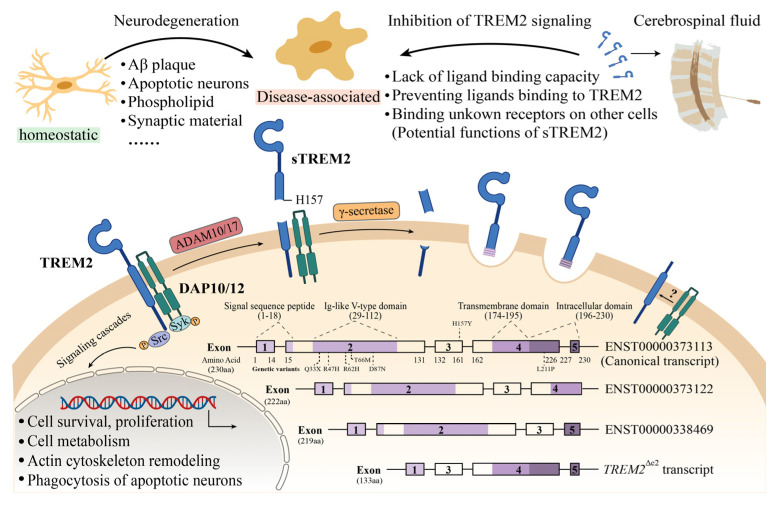
Schematic diagrams of soluble *TREM2* (s*TREM2*) generation and function. Canonical transcript (ENST00000373113) consists of five exons; immunoglobulin-like (Ig-like) domain and transmembrane (TM) domain are present in exon 2 and exon 4, respectively. The locations of some variants (p.Q33X, p.R47H, p.R62H, p.T66M, p.D87N, p.H157Y and p.L211P) are shown in the canonical *TREM2* domain. ADAM10/ADAM17 sheddase cleave *TREM2* receptor on histidine 157, contributing to the liberation of s*TREM2*. The C-terminal fragment is further cut by γ-secretase from the membrane. ENST00000373122 transcript lacks exon 5, and ENST00000338469 transcript lacks exon 4. *TREM2*^Δe2^ transcript lacks exon 2 and retains all other exons. Upon *TREM2*–ligand interaction, two tyrosine residues in ITAM of DAP12 are phosphorylated followed by recruiting SYK that initiates activation of a cascade of signaling events, such as cell survival and proliferation, cell metabolism, actin cytoskeleton remodeling, and phagocytosis of apoptotic neurons. s*TREM2* affects ligand binding capacity, prevents ligand binding to *TREM2*, and binds unknown receptors on other cells, thereby inhibiting *TREM2* signaling.

Four major transcripts of *TREM2* have been reported in human brains [17,18]. The N-terminus extracellular domain of the protein product of canonical *TREM2* (ENST00000373113) can be cleaved by a disintegrin and metalloproteinase 10 and/or 17 (ADAM10/17) at histone 157 and serine 158. This yields soluble *TREM2* (s*TREM2*). s*TREM2* is also generated through the alternative spliced *TREM2* isoform (ENST00000338469) lacking exon 4 that contains the transmembrane domain. It is estimated that the translation of this transcript variant accounts for 25% of total s*TREM2* protein [19]. s*TREM2* can be measured in peripheral blood as well as extracellular fluid, such as the cerebrospinal fluid (Figure 1). Studies have investigated the concentrations of s*TREM2* in the CSF of AD patients at different clinical stages, including preclinical stage of AD (pre-AD), mild cognitive impairment (MCI) and AD dementia [20,21,22,23,24,25,26,27,28,29,30,31,32,33,34,35,36,37,38,39,40,41]. There is evidence suggesting disease stage-dependent changes of s*TREM2* in the CSF [39,42]. However, several reports showed conflicting results [21,26,28]. Therefore, pooled data of a series of clinical trials are needed to determine the dynamic changes of s*TREM2* in AD patients. In this study, we performed a systematic review and meta-analysis of observational studies to assess the association between CSF s*TREM2* levels and AD. Because alterations of CSF s*TREM2* levels were also reported in other NDDs, such as PD, multiple sclerosis (MS), FTD and dementia with Lewy bodies (DLB) [36,38], albeit not as extensive as in AD, we extended our analysis to these conditions.

## 2. Methods

This meta-analysis was conducted strictly in accordance with the Meta-analysis of Observational Studies in Epidemiology (MOOSE) guidelines and Preferred Reporting Items for Systematic Reviews and Meta-Analyses (PRISMA) guidelines [43,44]. The MOOSE checklist is included in Table S1 in the Supplement.

### 2.1. Search Strategy

CSF s*TREM2* was first identified and reported in 2008 [36]. Full text articles written in English and published from 1 January 2008, to 24 February 2022, and collected from PubMed, Embase, Web of Science and Cochrane Library were searched using key words “cerebrospinal fluid” and “soluble *TREM2*”. The complete search used for PubMed was: (“cerebrospinal fluid” OR “CSF”) AND (“soluble *TREM2*” OR “s*TREM2*” OR “sTREM-2”). The articles retrieved were further manually evaluated to identify additional related studies. Two investigators conducted the literature searches independently (W.C.Z. and Y.T.Z.).

### 2.2. Eligibility Criteria

All included studies in the meta-analysis met the following criteria: (1) study design: observational studies including cohort, case–control or cross-sectional studies; (2) population: patients with NDDs and healthy controls; (3) CSF s*TREM2* levels were measured and reported as mean plus standard deviation (or standard error); (4) NDDs were diagnosed according to established diagnostic criteria (e.g., National Institute on Aging-Alzheimer’s Association [NIA-AA]); and (5) outcome variable: CSF s*TREM2* levels. The exclusion criteria were (1) studies of low-quality using Newcastle–Ottawa Scale (NOS) and Agency for Healthcare Research and Quality (AHRQ) tools; (2) duplicate publications; (3) reviews, comments, letters or conference abstracts; and (4) animal studies or studies on cadaver subjects.

### 2.3. Data Extraction and Quality Assessment

The relevant articles were reviewed and assessed by two investigators (W.C.Z. and Y.T.Z.) independently. The following information was extracted from each eligible study: basic data (first author, publication year, location, study design, measurement method of s*TREM2*, diagnostic criteria, disease type, number of participants, mean age and gender distribution) and outcome (CSF s*TREM2* levels). The NOS was applicable to assess the quality of the case–control studies with scores ranged from 0 to 9. A study with an NOS score ≥ 6 was considered as high quality [45]. The quality of the cross-sectional studies was evaluated using an 11-item checklist of AHRQ. A study with an AHRQ score ≥ 5 was considered as high quality (http://www.ncbi.nlm.nih.gov/books/NBK35156/ (accessed on 26 April 2022)).

### 2.4. Statistical Analysis

The standardized mean difference (SMD) and 95% confidence interval (CI) were calculated for the continuous variable (CSF s*TREM2* levels). Statistical heterogeneity was tested using the Cochran Q test and *I^2^* test. If heterogeneity was substantial (*p* < 0.05, *I*^2^ > 50%), sensitivity analysis was performed to identify the sources of the heterogeneity. If the heterogeneity could not be eliminated, a random-effect model was then used. Subgroup analysis was carried out based on the measurement method of CSF s*TREM2*. We conducted meta-regression to assess the impact of study characteristics on the CSF s*TREM2* levels. The explanatory variables included mean age, gender ratio, diagnostic criteria of NDDs. Publication bias was assessed using Begg’s and Egger’s linear regression tests. A *p* value greater than 0.5 was defined as no publication bias. For all statistical analyses, *p* < 0.05 was considered as statistically significant. StataSE 12.0 statistical software (Stata Corp., College Station, TX, USA) was used for all statistical analyses.

## 3. Results

### 3.1. Study Characteristics and Quality Assessment

**Figure 2:**
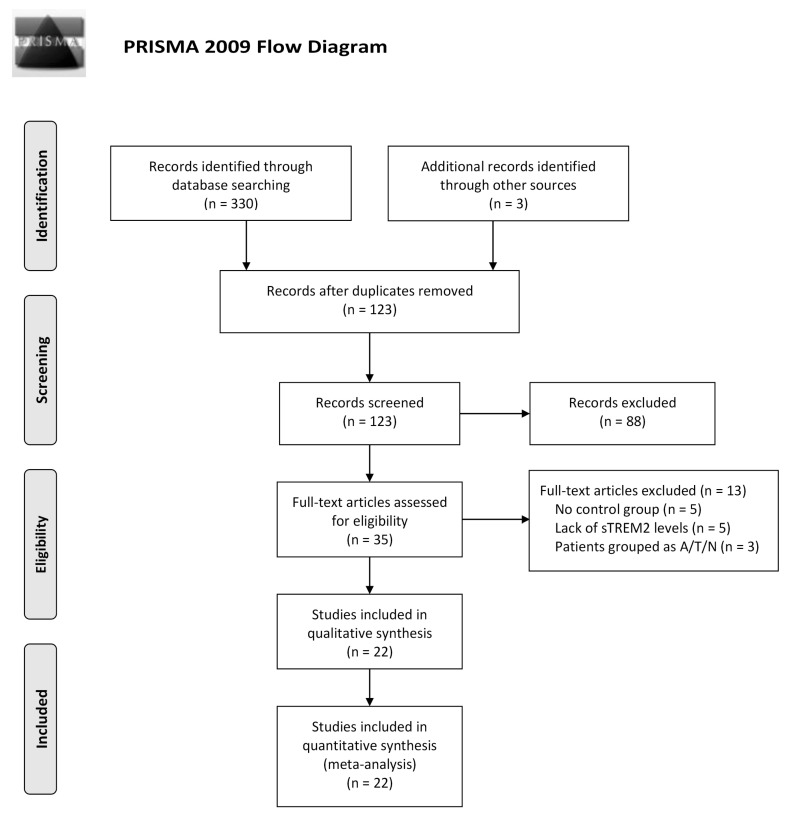
Flow diagram for identifying eligible studies.

We identified 333 studies, of which 22 (with data for 5716 participants) were included in our analysis. The flow diagram of the search procedure is shown in Figure 2 and the characteristics of the included studies are described in Table 1 [20,21,22,23,24,25,26,27,28,29,30,31,32,33,34,35,36,37,38,39,40,41]. All studies were published between 2008 and 2021. The mean age of patients with AD continuum (pre-AD, MCI and AD) was 70.8 (60.4–79.0) years and the mean age of other NDDs (e.g., PD, FTD, MS and DLB) was 63.5 (38.0–76.5) years. Among these studies, fourteen were carried out in Europe [20,21,22,26,27,28,29,30,33,34,37,38,39,41], three in Asia [31,32,35], and two in the United States of America [36,40]. Three studies used Alzheimer’s Disease Neuroimaging Initiative (ADNI) databases [23,24,25]. Regarding the measurement methods of CSF s*TREM2* levels, thirteen studies used the enzyme-linked immunosorbent assay (ELISA) [23,25,27,29,30,31,32,33,34,35,36,37,38], six studies used the Meso-Scale Discovery (MSD) [20,22,24,26,39,41], three studies used other methods including NeuroToolKit, ultra-performance liquid chromatography–tandem mass spectrometer (UPLC–MS) [21,28,40]. Details of the risk of bias assessment are provided in Tables S2 and S3 in the Supplement.

### 3.2. CSF sTREM2 Levels in AD Continuum Cohorts

**Figure 3:**
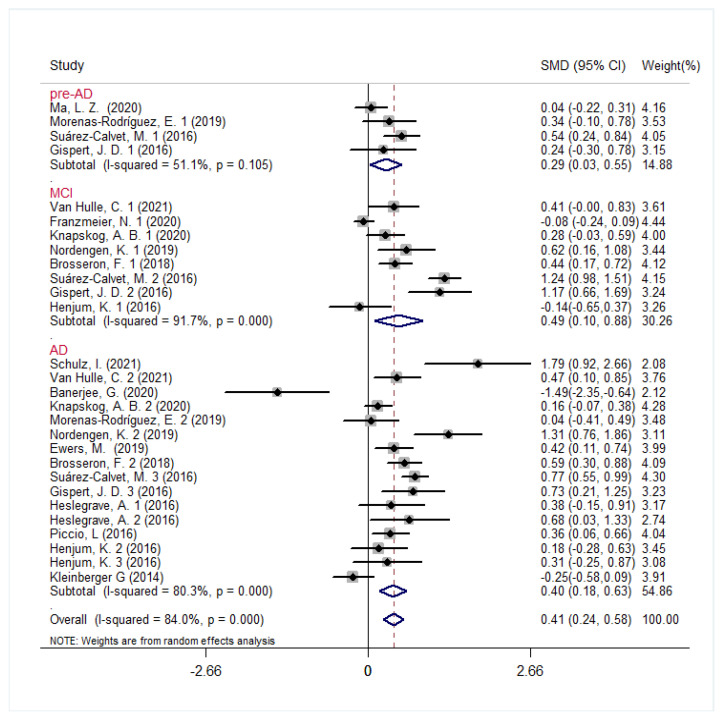
Comparison of CSF s*TREM2* between the whole AD continuum (pre-AD, MCI and AD dementia) and control groups.

Fifteen studies that included 193 pre-AD, 838 MCI, 1026 AD patients and 1613 controls measured the CSF s*TREM2* levels, for which a random-effect model was used (*p* < 0.001, *I*^2^ = 84%). There was a significant increase in CSF s*TREM2* levels in the whole AD continuum group as compared to controls (SMD, 0.41 [95% CI: 0.24, 0.58], *p* < 0.001, Figure 3). Subsequent analyses indicated that the CSF s*TREM2* levels of each disease stage (pre-AD, MCI, and AD) were higher compared with controls. The MCI group displayed the largest effect size (SMD, 0.49 [95% CI: 0.10, 0.88], *p* = 0.014, Figure 3), followed by the AD group (SMD, 0.40 [95% CI: 0.18, 0.63], *p* < 0.001, Figure 3). The increase in s*TREM2* levels in the pre-AD group was the lowest (SMD, 0.29 [95% CI: 0.03, 0.55], *p* = 0.031, Figure 3).

### 3.3. CSF sTREM2 Levels in Other NDDs

**Figure 4:**
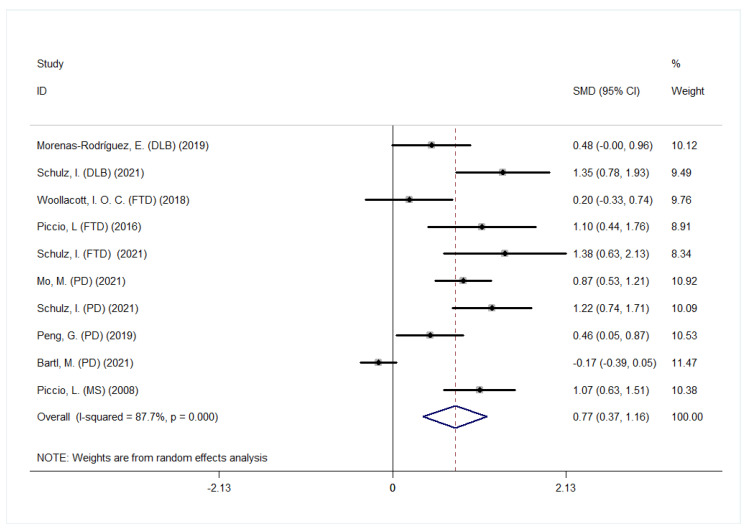
Comparison of CSF s*TREM2* between other NDDs and control groups.

Eight studies that included 752 patients with other NDDs (538 PD, 89 FTD, 73 DLB and 52 MS) and 449 controls measured the CSF s*TREM2* levels, for which a random-effect model was used (*p* < 0.001, *I*^2^ = 87.7%). Among these, 4 reported s*TREM2* levels in PD patients, 3 in FTD, 2 in DLB and 1 in MS. Except for one study on the association between s*TREM2* and PD by Bartl, M. et al., the rest of the studies reported a higher level of CSF s*TREM2* than controls (Figure 4). Collectively, NDDs showed significantly increased levels of CSF s*TREM2* compared with control group (SMD, 0.77 [95% CI: 0.37, 1.16], *p* < 0.001, Figure 4). However, one should be aware that each of the three NDDs has its unique pathological and clinical features. In addition, we combined AD with the rest of NDD studies, and performed a pooled analysis. The results also suggested that CSF s*TREM2* was elevated in NDDs with a significant between-study heterogeneity (*p* < 0.001, *I*^2^ = 87.4%) (Figure S1 in the Supplement).

### 3.4. Heterogeneity Analysis and Subgroup Analysis

The pooled results of CSF s*TREM2* level were relatively reliable in the pre-AD cohort (*I*^2^ = 51.1%, *p* = 0.105), while a significant between-study heterogeneity was observed in the MCI group (*I*^2^ = 91.7%, *p* < 0.001). Therefore, we conducted sensitivity analysis and then subgroup analysis based on the measurement methods of s*TREM2* (Figure S2 in the Supplement). We excluded studies from Franzmeier, N. et al. and Suárez-Calvet, M. et al. based on the results of sensitivity analysis, and the *I^2^* value fell from 91.7% to 65%. There was no significant between-study heterogeneity in the ELISA subgroup (*p* = 0.096, *I*^2^ = 57.3%, Table 2). Compared with the control group, the levels of CSF s*TREM2* in the non-ELISA subgroup were statistically higher (SMD, 0.63 [95% CI: 0.22, 1.05], *p* = 0.003, Table 2), while there was no statistical increase in the ELISA subgroup.

**Figure 5:**
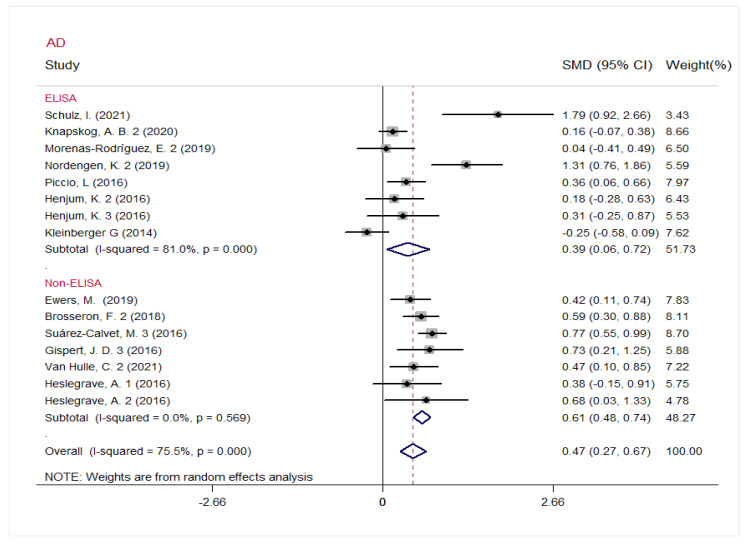
Subgroup analysis of CSF s*TREM2* in AD dementia cohort based on measurement methods (ELISA and non-ELISA) of s*TREM2*.

Subsequently, heterogeneity analysis and subgroup analysis were performed in the AD group. The pooled results showed that the *I^2^* value fell from 74.6% to 0% (*p* = 0.569, Figure 5) in the non-ELISA subgroup (SMD, 0.61 [95% CI: 0.48, 0.74], *p* < 0.001), while there exists significant between-study heterogeneity in the ELISA subgroup (*p* < 0.001, *I*^2^ = 81.0%, Figure 5). We then conducted Galbraith plot to identify the sources of the heterogeneity (Figure S3A in the Supplement). The studies that were distributed outside of parallel lines were excluded. The pooled results showed that the *I*^2^ value fell from 81.0% to 0% (*p* = 0.758, Figure S3B in the Supplement) in the ELISA subgroup, and the CSF s*TREM2* level were higher in the AD group (SMD, 0.21 [95% CI: 0.06, 0.36], *p* = 0.006).

Heterogeneity also existed in CSF s*TREM2* levels of other NDDs (*I*^2^ = 87.7%). Therefore, sensitivity analyses were performed to identify the sources (Figure S4A in the Supplement). In all eight studies that reported CSF s*TREM2* levels, two studies by Bartl, M. et al. (NeuroToolKit) and Woollacott, I. O. C. et al. (MSD) used the non-ELISA method to measure s*TREM2* levels. We excluded these studies and found that the *I*^2^ value fell from 87.7% to 49.8%. Compared with the control group, the CSF s*TREM2* level of NDDs (ELISA) was significantly higher (SMD, 0.94 [95% CI: 0.70, 1.19], *p* < 0.001, Figure S4B in the Supplement).

### 3.5. Meta-Regression and Publication Bias

We performed meta-regression on 16 studies comparing CSF s*TREM2* levels between AD patients and control group, and 10 studies between other NDDs and control group. Our results showed that mean age, gender ratio and diagnostic criteria of AD and other NDD groups had no significant impact (*p* > 0.05) on the effect sizes of the differences of CSF s*TREM2* levels (Table S4 in the Supplement). The number of studies regarding pre-AD and MCI was not sufficient for meta-regression.

Begg’s and Egger’s tests were carried out to assess the potential publication bias (*t* = 1.18, *p* = 0.248), however, none was found.

## 4. Discussion

CSF s*TREM2* level has been extensively investigated in several NDDs, especially in AD [21,32,38,40]. Our pooled results showed that CSF s*TREM2* is reliably elevated in NDDs, which is consistent with microglia activation observed in these conditions, suggesting that it is a potential biomarker for NDDs. In AD, dynamic release of s*TREM2* is influenced by disease stage. AD continuum develops with hallmark pathological changes, such as amyloid beta deposition and accumulation of hyperphosphorylated tau (p-tau) protein. These pathophysiological changes usually begin many years prior to the spectrum of AD spans (pre-AD, MCI and AD dementia) from clinically asymptomatic to severely impaired [46,47,48,49]. Previous studies reported the correlations between hallmarks of AD pathology and increased s*TREM2* levels. The increase in CSF s*TREM2* occurs before the onset of symptoms, but after amyloidosis and neuronal injury [42]. Total and phosphorylated tau (T-tau and p-tau) in CSF are markers of neurodegeneration subsequent to the development of Aβ deposition. Several studies found the association between levels of s*TREM2* in CSF and T-tau and p-tau, but not with Aβ in AD [27,28,30,37]. Our analysis supported their observations. Levels of CSF s*TREM2* peaked at early symptomatic stage, and remained high at AD dementia, without signs of further increase.

The concentration of s*TREM2* in CSF is affected not only by AD pathology, but also by genetic variations of *TREM2*. For example, carriers of the R47H (rs75932628) mutation of *TREM2* have higher level of CSF s*TREM2* than non-carriers, they display strong association with AD, exhibit early onset and rapid progression of cognitive impairment [11,37,50,51]. On the other hand, carriers of the p.Q33X or p.T66M mutations have lower s*TREM2* concentrations than others [37]. The p.H157Y variant of *TREM2* enhances ectodomain shedding, thereby reducing cell surface expression of *TREM2* [52,53]. The studies we included in this analysis did not have the information on *TREM2* genetic variations of the participants. However, one should be aware of the effect of *TREM2* mutation on the secretion of s*TREM2*.

Our analysis also suggested that CSF s*TREM2* level increased significantly in NDDs other than AD. However, this finding should be interpreted with caution since each NDD has its own pathological and clinical feature, and studies that reported the association between s*TREM2* and other NDDs were limited. On the other hand, *TREM2* variants are found to be associated with increased risk for these neurological conditions, and CSF s*TREM2* was observed to correlate with neuronal injury markers [21,38,41]. Further studies are needed to investigate the molecular mechanism of s*TREM2* in other NDDs.

While majority of the studies used ELISA to measure s*TREM2* level, we also found several studies which quantitated s*TREM2* using other methods. Our analysis showed that the pooled results from non-ELISA studies had low heterogeneity and the concentration of s*TREM2* was significantly higher than the ELISA group. The non-ELISA group included studies using several different methods, such as MSD, NeuroToolKit and UPLC–MS. Our analysis suggested that these methods are also reliable approaches in measuring s*TREM2*. On the other hand, multiple factors may cause variations in ELISA results, especially the choice of antibody and the handling of CSF samples. The antibodies used for sTREM detection were Polyclonal Goat IgG (His19-Ser174) and Monoclonal Rabbit IgG (Met1-Ser174) in the included studies. The peptide sequence corresponds to cDNA sequence of exons 1, 2 and 3. Therefore, the lack of exon 4 should not affect the detection.

There are several strengths in our meta-analysis. Compared with previous ones, we included more high-quality studies and investigated the associations between s*TREM2* and NDDs other than AD [54,55]. In addition, we performed an in-depth analysis on AD continuum and attempted to elucidate the underlying pathological mechanisms by which the results are connected. Furthermore, we identified the sources of heterogeneity, which made our pooled results reliable.

A limitation of this analysis is that most available studies to date are observational. The association between s*TREM2* and NDDs needs to be investigated in prospective longitudinal studies that include patients with preclinical NDDs to understand the microglial activation in response to the progression of neuronal injury. Furthermore, the treatment history of the participant could also affect the CSF s*TREM2* concentration. However, detailed information was not available in most of the studies included in this analysis. Finally, since this study was designed to include only those which reported the mean concentration of CSF s*TREM2*, studies reporting median s*TREM2* level were excluded.

## 5. Conclusions

In conclusion, our pooled data confirmed the robust association between CSF s*TREM2* level and NDDs, and suggested that CSF s*TREM2* is a dynamic biomarker for microglial activation during neuroinflammation. In future research, it is essential to investigate the interrelationship between the levels of s*TREM2* and disease pathology and genetic variants, and further identify the clinical implication of higher s*TREM2* in NDDs patients.

## Figures and Tables

**Table 1:** Baseline characteristics of included studies.

First Author	Year	Location	Design	Method	Diagnostic Criteria	Disease	Case				Control			
							n	Age, y, Mean (SD)	No. of Female (%)	s*TREM2* (ng/mL)	n	Age, y, Mean (SD)	No. of Female (%)	s*TREM2* (ng/mL)
Schulz, I.	2021	Germany	CS	ELISA	NINDS-ADRDA	AD	11	74.27 (4.64)	5 (45)	7.750 (3.815)	20	68.75 (6.38)	6 (30)	3.218 (1.463)
Van Hulle, C.	2021	US	CS	NTK	NIA-AA	MCI	33	74.1 (7.6)	13 (39)	9.75 (3.68)	70	69.1 (6.6)	45 (64)	8.51 (2.63)
						AD	46	72.3 (8.0)	18 (39)	9.95 (3.57)				
Franzmeier, N.	2020	ADNI	CS	ELISA	NINCDS-ADRDA	MCI	414	71.82 (7.45)	170 (41)	4.095 (2.105)	221	74.25 (6.08)	106 (48)	4.256 (2.184)
						AD	73	74.17 (8.37)	35 (48)	4.371 (2.194)				
Banerjee, G.	2020	UK	CC	MSD	NIA-AA	AD	20	62.5 (4.1)	11 (55)	6.669 (0.664)	10	62.2 (5.4)	5 (50)	7.960 (1.183)
Knapskog, A. B.	2020	Norway	CS	ELISA	NIA-AA	MCI	62	71.0 (5.4)	36 (58.1)	9.9 (4.5)	113	72.3 (6.0)	54 (47.8)	8.8 (3.6)
						AD	237	70.1 (6.8)	135 (57)	9.5 (4.8)				
Ma, L. Z.	2020	China	CS	ELISA	NIA-AA	pre-AD stage 1	148	60.39 (10.41)	63 (42.6)	15.12 (6.40)	242	60.81 (9.95)	96 (39.7)	18 (6.34)
						pre-AD stage 2	70	64.19 (11.09)	31 (42.3)	18.28 (7.54)				
Morenas-Rodríguez, E.	2019	Spain	CC	ELISA	NIA-AA	pre-AD	53	72.3 (6.3)	32 (60.4)	5 (2.4) (No. = 41)	44	67.4 (5.1)	25 (56.8)	4.2 (2.3) (No. = 40)
						AD	50	74.6 (5.6)	31 (62)	4.3 (2.2) (No. = 36)				
Nordengen, K.	2019	Norway	CC	ELISA	NIA-AA	MCI	40	66.6 (7.4)	23 (57)	4.0 (1.8)	36	61.1 (9.2)	19 (53)	3.1 (0.9)
						AD	27	67.6 (5.2)	13 (48)	4.8 (1.7)				
Deming, Y.	2019	ADNI	CS	ELISA	NINCDS-ADRDA	EMCI	183	71.23 (7.39)	77 (42.1)	3.74 (2.07)	169	74.47 (5.85)	80 (47.3)	3.99 (1.92)
						LMCI	221	73.06 (7.41)	91 (41.2)	3.92 (1.83)				
						AD	172	74.39 (8.56)	74 (43.0)	4.02 (1.95)				
Ewers, M.	2019	ADNI	CS	MSD	NIA-AA	MCI	184	72.9 (7.11)	77 (41.8)	4.452 (2.518)	100	72.8 (5.36)	45 (45)	3.762 (1.841)
						AD	66	73.6 (8.51)	32 (48.5)	4.608 (2.201)				
Brosseron, F.	2018	Germany	CC	MSD	NIA-AA	MCI	130	71 (8)	65 (50)	4.07 (2.54)	85	67 (11)	65 (76)	2.99 (2.27)
						AD	116	74 (8)	45 (39)	4.32 (2.24)				
Suárez-Calvet, M.	2016	Multi-center	CC	MSD	NIA-AA	pre-AD	63	70.8 (11)	38 (60)	4.09 (2.7)	150	62.4 (11)	89 (59)	3.07 (1.4)
						MCI	111	74.3 (9)	67 (60)	5.98 (3.2)				
						AD	200	73.8 (10)	124 (62)	5.33 (3.7)				
Gispert, J. D	2016	Spain	CC	MSD	NIA-AA	pre-AD	19	68.53 (7.93)	13 (68.42)	2.70 (1.47)	45	60.98 (6.83)	28 (63.04)	2.40 (1.14)
						MCI	27	70.30 (7.35)	15 (55.56)	4.16 (1.97)				
						AD	23	66.78 (9.75)	16 (69.57)	3.34 (1.53)				
Heslegrave, A.	2016	UK Sweden	CC	UPLC-MS	IWG2	AD	37	70.51 (7.5)	19 (53)	0.231 (0.098)	22	69.2 (8.0)	10(45)	0.196 (0.081)
						AD	24	64.3 (6.8)	13 (54)	0.231 (0.097)	16	55.6 (9.7)	9 (56)	0.173 (0.065)
Piccio, L	2016	Italy/US	CC	ELISA	NINCDS-ADRDA	AD	73	76.6 (5.2)	36 (49)	1.028 (0.582)	107	70.2 (8.5)	57 (53)	0.832 (0.508)
Henjum, K	2016	Norway Sweden	CC	ELISA	NIA-AA	MCI	21	67.0 (5.0)	12 (57)	4.10 (2.59)	50	66.0 (9.0)	25 (50)	4.40 (2.00)
						AD	29	68.0 (4.8)	13 (45)	4.80 (2.67)				
					NINCDS-ADRDA	AD	25	79.0 (6.3)	18 (72)	3.80 (2.20)	25	62.0 (9.3)	17 (68)	3.20 (1.63)
Kleinberger G	2014	Germany	CS	ELISA	NINCDS-ADRDA	AD	56	70.4 (8.9)	38 (68)	0.3087 (0.191) (RU)	88	60.7 (9.5)	55 (63)	0.3834 (0.174)
Schulz, I.	2021	Germany	CS	ELISA	UKPDSBB	PD	151	69.36 (9.55)	51 (34)	6.494 (2.794)	20	68.75 (6.38)	6 (30)	3.218 (1.463)
					FTDC	FTD	15	70.80 (5.58)	5 (33)	6.486 (3.210)				
					DLB consensus	DLB	45	70.51 (6.51)	14 (31)	6.375 (2.620)				
Bartl, M.	2021	Multi-center	CC	NTK	UKPDSBB	PD	252	61 (9.8)	87 (34.5)	6.9 (2.2)	115	62 (11)	41 (35.7)	7.3 (2.7)
Mo, M.	2021	China	CC	ELISA	UKPDSBB	PD	80	63.59 (8.50)	32 (40)	0.419 (0.182)	65	62.49 (6.90)	26 (40)	0.290 (0.090)
Peng, G.	2019	China	CC	ELISA	UKPDSBB	PD	55	59.8 (8.9)	28 (51)	0.4331 (0.0247)	40	55.6 (13.4)	19 (47.5)	0.2752 (0.0179)
Morenas-Rodríguez, E.	2019	Spain	CC	ELISA	DLB consensus	DLB	37	76.5 (5)	20 (54.1)	5.3 (2.3) (No. = 28)	44	67.4 (5.1)	25 (56.8)	4.2 (2.3) (No. = 40)
Woollacott, I. O. C.	2018	UK	CC	MSD	FTDC	FTD	64	64.6 (6.5)	19 (29.7)	7.4 (3.2)	17	63.7 (6.4)	11 (64.7)	6.8 (1.6)
Piccio, L	2016	Italy/US	CC	ELISA	FTDC	FTD	10	-	-	1.396 (0.563)	107	70.2 (8.5)	57 (53)	0.832 (0.508)
Piccio, L.	2008	US	CC	ELISA	McDonald criteria	MS	52	54 (9)	31 (59.6)	0.9 (0.55)	41	44 (15)	31 (76)	0.43 (0.23)

Abbreviations: CS, cross-sectional; CC, case-control; NINCDS-ADRDA, National Institute of Neurological and Communicative Disorders and Stroke and the Alzheimer’s Disease and Related Disorders Association; NIA-AA, National Institute on Aging-Alzheimer’s Association; NTK, NeuroToolKit; ADNI, Alzheimer’s Disease Neuroimaging Initiative; MSD, Meso-Scale Discovery; IWG2, the revised proposed International Working Group; UPLC–MS, ultra-performance liquid chromatography–tandem mass spectrometer; FTDC, International consensus criteria for behavioral variant FTD; pre-AD, preclinical stage of Alzheimer’s disease; MCI, mild cognitive impairment; EMCI, early MCI; LMCI, late MCI; AD, Alzheimer’s disease; PD, Parkinson’s disease; FTD, frontotemporal dementia; DLB, dementia with Lewy bodies; MS, multiple sclerosis; RU, Relative Units; -, not available.

**Table 2:** Subgroup analysis of CSF s*TREM2* levels in NDDs based on measurement methods.

Diseases	No. of Trials	Subgroup	Overall Effect			Heterogeneity	
			SMD	95% CI	*p* Value	*p* of *I*^2^	*I* ^2^
MCI	6	Overall	0.45	0.18~0.73	0.001	0.014	65.0%
	3	ELISA	0.27	−0.10~0.64	0.157	0.096	57.3%
	3	Non-ELISA	0.63	0.22~1.05	0.003	0.037	69.6%
AD	15	Overall	0.47	0.27~0.67	0.000	0.000	75.5%
	8	ELISA	0.39	0.06~0.72	0.020	0.000	81.0%
	7	Non-ELISA	0.61	0.48~0.74	0.000	0.569	0.0%
Other NDDs	8	ELISA	0.94	0.70~1.19	0.000	0.052	49.8%

## Data Availability

The data that support the findings of this study are available from the corresponding author upon reasonable request.

## Supplementary Materials

The following supporting information can be downloaded at: https://www.mdpi.com/article/10.3390/jcm12103589/s1, Table S1. MOOSE Checklist; Table S2. Quality Assessment of the Case-control Studies using NOS; Table S3. Quality Assessment of the Cross-sectional Studies using AHRQ; Table S4. Meta-regression Analysis of Baseline Characteristics; Figure S1. Comparison of CSF s*TREM2* between NDDs and control groups; Figure S2. Sensitivity and subgroup analysis of CSF s*TREM2* levels in MCI: (A) The influence of each trial for CSF s*TREM2* levels of the meta-analysis; (B) Subgroup of the CSF s*TREM2* levels based on measurement methods; Figure S3. Heterogeneity analysis of CSF s*TREM2* levels in AD: (A) The Galbraith plot of CSF s*TREM2* levels in this meta-analysis; (B) comparison of the CSF s*TREM2* levels measured by ELISA; Figure S4. Sensitivity analysis of CSF s*TREM2* levels in NDDs: (A) The influence of each trial for CSF s*TREM2* levels of the meta-analysis; (B) comparison of the CSF s*TREM2* levels measured by ELISA.
