# Supplementary material for: Association between Cerebrospinal Fluid Soluble TREM2, Alzheimer’s Disease and Other Neurodegenerative Diseases
Source: J Clin Med. 2023 May 22;12(10):3589. doi: 10.3390/jcm12103589 (PMC10219449; doi:10.3390/jcm12103589)
Supplement: Supplementary file 1 [file jcm-12-03589-s001.zip › jcm-2032669-supplementary.pdf]

Table S1. MOOSE Checklist

Criteria		Brief description of how the criteria were handled in the meta-analysis
**Reporting of background should include**		
√	Problem definition	Heterozygous rare variants in triggering receptor expressed on myeloid cells 2 (*TREM2*) are associated with increased risk for neurodegenerative diseases (NDDs). Cerebrospinal fluid (CSF) soluble TREM2 (sTREM2) is a potential biomarker and therapy target for NDDs.
√	Hypothesis statement	CSF sTREM2 increases in a disease stage-dependent fashion in AD. sTREM2 also increases in other NDDs compared to controls as a biomarker for microglial activation.
√	Description of study outcomes	CSF sTREM2 levels
√	Type of exposure or intervention used	NA.
√	Type of study designs used	Observational studies: case-control and cross-sectional studies
√	Study population	patients with NDDs and healthy controls without a history of NDDs
**Reporting of search strategy should include**		
-	Qualifications of searchers	—
√	Search strategy, including time period included in the synthesis and keywords	We used the following key words: "cerebrospinal fluid" and "soluble TREM2" to search relevant studies published from January 1st, 2008, to February 24th, 2022.
√	Databases and registries searched	PubMed, Embase, Web of Science and Cochrane Library
√	Search software used, name and version, including special features	EndNote X9 was used to merge retrieved citations and eliminate duplications.
√	Use of hand searching	We hand-searched bibliographies of retrieved papers for additional references.
√	List of citations located and those excluded, including justifications	Details of the literature search process are outlined in the flow chart. The citation list is available upon request.
√	Method of addressing articles published in languages other than English	We included studies published in English language.
√	Method of handling abstracts and unpublished studies	We extracted reported results from published articles.
√	Description of any contact with authors	We did not contact the authors for additional data.
**Reporting of methods should include**		
√	Description of relevance or appropriateness of studies assembled for assessing the hypothesis to be tested	Detailed inclusion and exclusion criteria were described in the methods section.
√	Rationale for the selection and coding of data	Data extracted from each of the studies were relevant to the study characteristics and results, including basic data and outcomes.
√	Assessment of confounding	We conducted sensitivity analysis, subgroup analysis, and meta-regression.
√	Assessment of study quality, including blinding of quality assessors; stratification or regression on possible predictors of study results	The Newcastle-Ottawa Scale (NOS) was applicable to assess the quality of the case-control studies. The quality of the cross-sectional studies was evaluated using an 11-item checklist of Agency for Healthcare Research and Quality (AHRQ).

√	Assessment of heterogeneity	Heterogeneity of the studies were evaluated using Cochrane's Q test of heterogeneity and I^2 statistic. We conducted sensitivity analysis, subgroup analysis and meta-regression analysis to identify the sources of heterogeneity.
√	Description of statistical methods in sufficient detail to be replicated	Description of methods of meta-analyses, sensitivity analyses, subgroup analysis, meta-regression, and assessment of publication bias, are detailed in the methods.
√	Provision of appropriate tables and graphics	We included 1 flow chart, 2 summary table, 2 table of quality assessment, 1 table of meta-regression, 6 forest plot of all studies, and 1 schematic diagram
**Reporting of results should include**		
√	Graph summarizing individual study estimates and overall estimate	Fig.2-4, eFig.1-3
√	Table giving descriptive information for each study included	Table 1
√	Results of sensitivity testing	In all eight studies that reported CSF sTREM2 levels of other NDDs, two studies by Bartl, M. et.al (NeuroToolKit) and Woollacott, I. O. C. (MSD) used non-ELISA method to measure sTREM2 levels. We excluded these studies and found that the I^2 value fell from 87.7% to 49.8%.
√	Indication of statistical uncertainty of findings	95% confidence intervals were presented with all summary estimates, I^2 values and results of sensitivity analyses
**Reporting of discussion should include**		
√	Quantitative assessment of bias	Sensitivity analyses indicate heterogeneity in strengths of the association due to most common biases in observational studies.
√	Justification for exclusion	Studies of low-quality using Newcastle-Ottawa Scale (NOS) and Agency for Healthcare Research and Quality (AHRQ) tools; duplicate publications; reviews, comments, letters or conference abstracts; animal studies or cadaver subjects.
√	Assessment of quality of included studies	NOS and AHRQ tools
**Reporting of conclusions should include**		
√	Consideration of alternative explanations for observed results	The variations in the strengths of association may be due to true population differences, or to differences in quality of studies.
√	Generalization of the conclusions	In conclusion, our pooled data confirmed the robust associations between CSF sTREM2 levels and NDDs, which suggested the CSF sTREM2 as a potential dynamic biomarker and therapy target for NDDs.
√	Guidelines for future research	In the future research, it is essential to investigate the interrelationships between the levels of sTREM2 and the alterations of pathology and genetic variants, further identifying the functions and clinical implications of sTREM2.
√	Disclosure of funding source	Ministry of Science and Technology of China (2018YFA0800801)<br>National Natural Science Foundation of China (81873679).

Table S2. Quality Assessment of the Case-control Studies using NOS

Case-control study	Year	Selection	Comparability	Exposure	Score	Quality assessment
Banerjee, G.	2020	★★	★★	★★	6	High
Morenas-Rodríguez, E.	2019	★★★	★★	★★★	8	High
Nordengen, K.	2019	★★	★★	★★★	7	High
Brosseron, F.	2018	★★	★★	★★★	7	High
Suárez-Calvet, M.	2016	★★★	★★	★★★	8	High
Gispert, J. D	2016	★★★	★★	★★	7	High
Heslegrave, A.	2016	★★	★★	★★	6	High
Piccio, L	2016	★★★	★★	★★	7	High
Henjum, K	2016	★★	★★	★★	6	High
Bartl, M.	2021	★★	★★	★★	6	High
Mo, M.	2021	★★	★★	★★★	7	High
Peng, G.	2019	★★	★★	★★★	7	High
Woollacott, I. O. C.	2018	★★	★★	★★	6	High
Piccio, L	2008	★★	★★	★★★	7	High

NOS: Newcastle-Ottawa Scale

Table S3. Quality Assessment of the Cross-sectional Studies using AHRQ

**Item**	**Schulz (2021)**	**Van Hulle (2021)**	**Franzmeier (2020)**	**Knapskog (2020)**	**Ma (2020)**	**Deming (2019)**	**Ewers (2019)**	**Kleinberger (2014)**
1) Define the source of information (survey, record review)	Yes	Yes	Yes	Yes	Yes	Yes	Yes	Yes
2) List inclusion and exclusion criteria for exposed and unexposed subjects (cases and controls) or refer to previous publications	Yes	Yes	Yes	Yes	Yes	Yes	Yes	Yes
3) Indicate time period used for identifying patients	No	No	No	Yes	No	No	No	No
4) Indicate whether or not subjects were consecutive if not population-based	Unclear	Unclear	Unclear	Yes	Unclear	Unclear	Unclear	Unclear
5) Indicate if evaluators of subjective components of study were masked to other aspects of the status of the participants	No	No	No	No	No	No	No	No
6) Describe any assessments undertaken for quality assurance purposes (e.g., test/retest of primary outcome measurements)	Yes	Yes	Yes	Yes	Yes	Yes	Yes	Yes
7) Explain any patient exclusions from analysis	Unclear	Yes	No	Yes	No	No	No	Unclear
8) Describe how confounding was assessed and/or controlled.	Yes	Yes	Yes	Yes	Yes	Yes	Yes	Yes
9) If applicable, explain how missing data were handled in the analysis	Unclear	Yes	Yes	Unclear	Yes	Yes	Yes	Unclear
10) Summarize patient response rates and completeness of data collection	No	Yes	Yes	Unclear	Yes	Yes	Yes	No
11) Clarify what follow-up, if any, was expected and the percentage of patients for which incomplete data or follow-up was obtained	Yes	No	Yes	No	Yes	Yes	Yes	Yes
**AHRQ Score**	5	7	7	7	7	7	7	5
**Quality assessment**	high	high	high	high	high	high	high	high

AHRQ: Agency for Healthcare Research and Quality

Table S4. Meta-regression Analysis of Baseline Characteristics

Variables	exp(b)	95% CI	P value
**AD**			
Mean age (year)	1.019087	0.9471863~1.096446	0.584
Sex ratio (%)	0.9835338	0.9543528~1.013607	0.253
Diagnostic criteria	1.057713	0.6787325~1.648304	0.788
**Other NDDs**			
Mean age (year)	1.020988	0.9401533~1.108773	0.560
Sex ratio (%)	1.003216	0.9534758~1.055552	0.882
**NDDs**			
Mean age (year)	0.9958138	0.9338874~1.061847	0.895

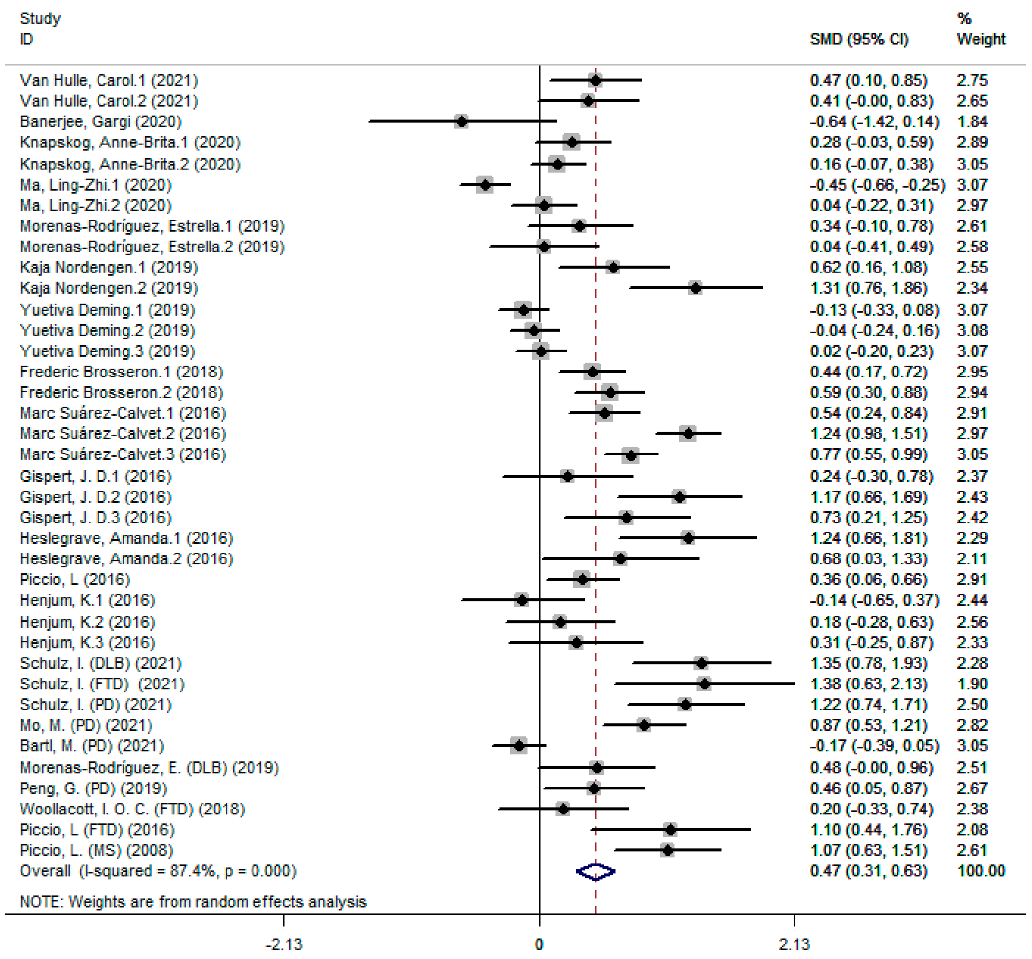

Figure S1. Comparison of CSF sTREM2 between NDDs and control groups.

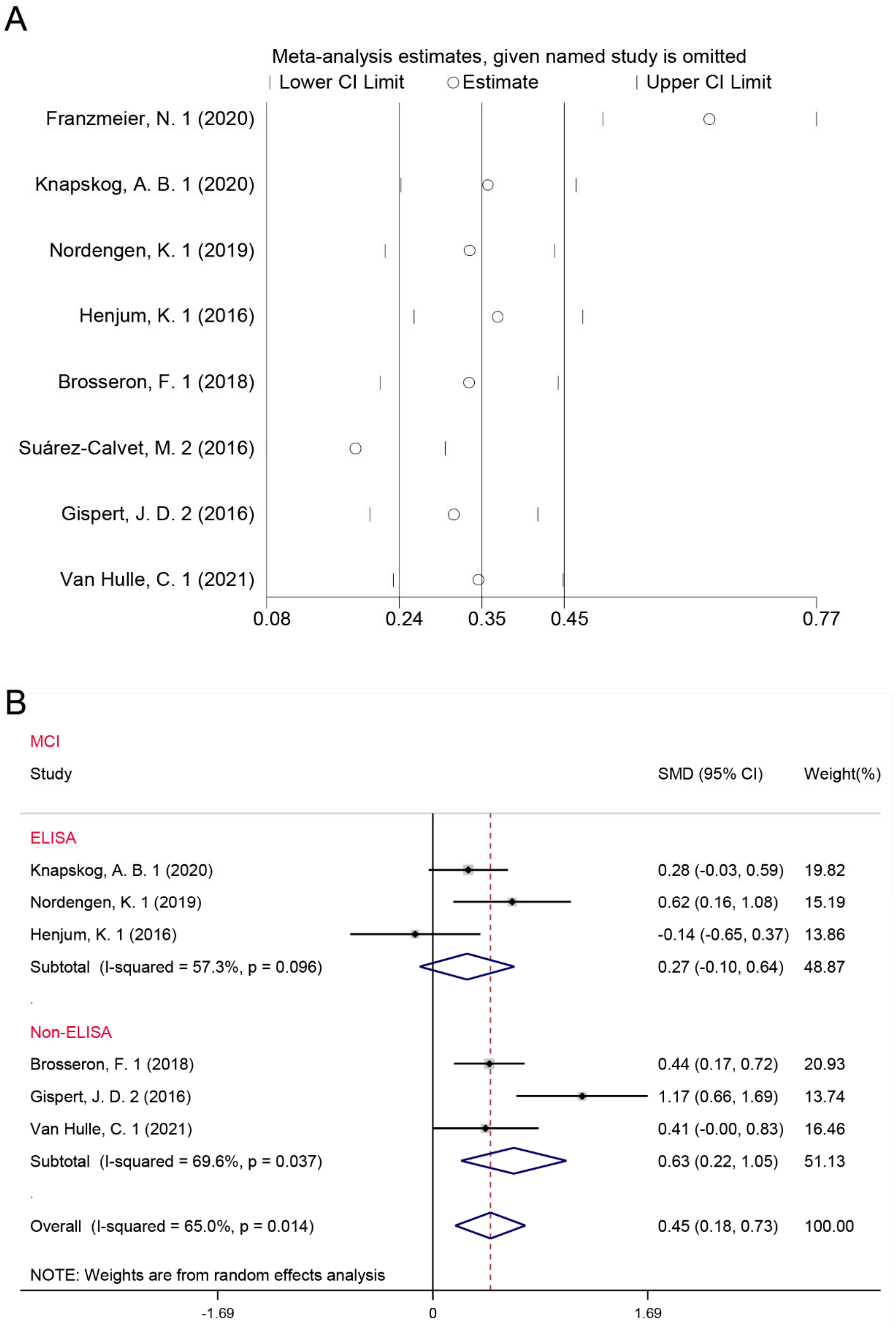

Figure S2. Sensitivity and subgroup analysis of CSF sTREM2 levels in MCI: A) The influence of each trial for CSF sTREM2 levels of the meta-analysis; B) Subgroup of the CSF sTREM2 levels based on measurement methods.

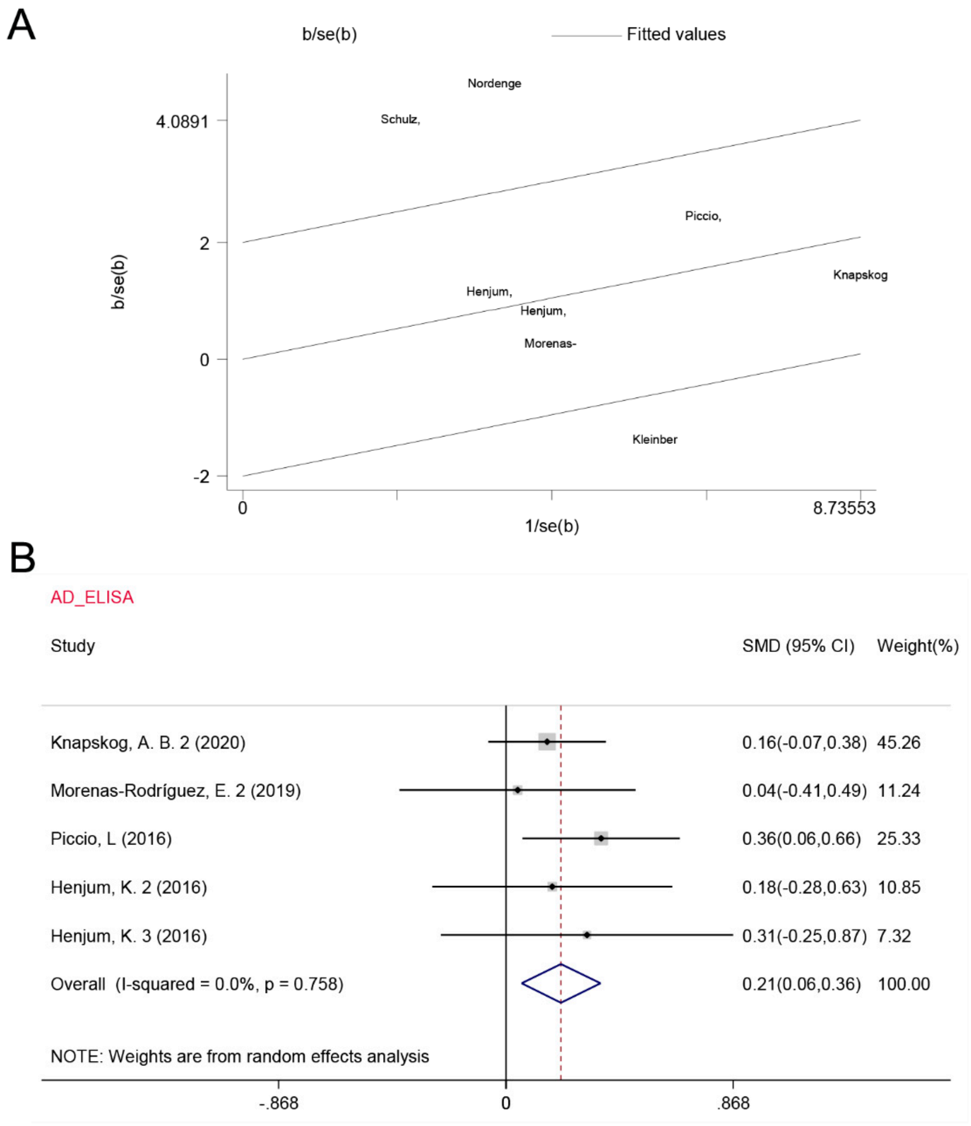

Figure S3. Heterogeneity analysis of CSF sTREM2 levels in AD: A) The Galbraith plot of CSF sTREM2 levels in this meta-analysis; B) comparison of the CSF sTREM2 levels measured by ELISA.

A

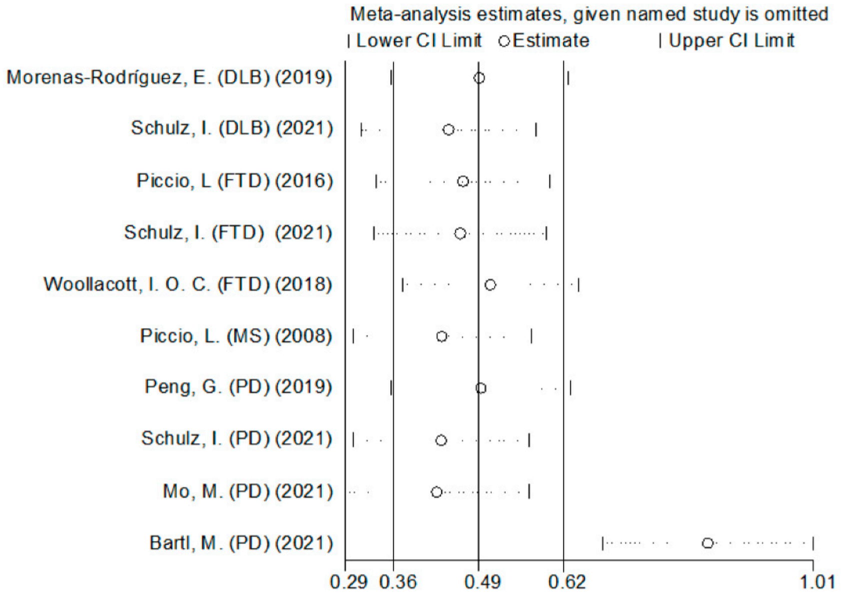

B

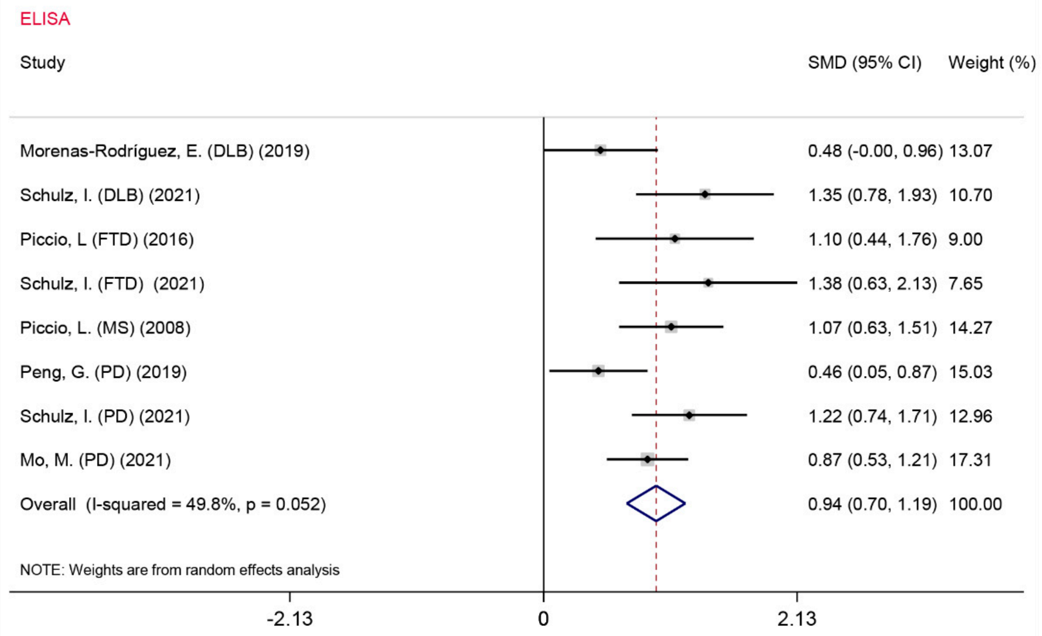

Figure S4. Sensitivity analysis of CSF sTREM2 levels in NDDs: A) The influence of each trial for CSF sTREM2 levels of the meta-analysis; B) comparison of the CSF sTREM2 levels measured by ELISA.
